# Supplementary material for: Critical factors for precise and efficient RNA cleavage by RNase Y in Staphylococcus aureus
Source: PLoS Genet. 2024 Aug 1;20(8):e1011349. doi: 10.1371/journal.pgen.1011349 (PMC11321564; doi:10.1371/journal.pgen.1011349)
Supplement: S1 Table — (DOCX) [file pgen.1011349.s001.docx]

### S1 Table. List of strains:

| **Strain**  **name** | **Description** | **Parent strain** | **Reference** |
| --- | --- | --- | --- |
| *Staphylococcus aureus* strains | | | |
| PR01 (WT) | Derivative of clinical strain SA564, Δ*pyrFE* and restriction deficient | SA564 | (1) |
| PR01-02 (ΔY) | PR01 with a Δ*rny* deletion (*rny* is also known as *cvfA*) | PR01 | (1) |
| L2ALS01 (BsY) | *S. aureus* RNase Y ORF (*rny* gene) replaced with *B. subtilis* RNase Y ORF (C-terminally tagged with Streptavidine-Flag- His6) | PR01 | This work |
| L2ALS11 | pBsCgg | L2ALS01 | This work |
| L2ALS12 | pBsCgg | PR01 | This work |
| L2ALS13 | pBsCgg | PR01-02 | This work |
| L2ALS22 | pSaGap[ΔIII] | PR01 | This work |
| L2ALS23 | pSaGap[ΔIII] | PR01-02 | This work |
| L2ALS32 | pSaGap[ΔI] | PR01 | This work |
| L2ALS33 | pSaGap[ΔI] | PR01-02 | This work |
| L2ALS37 | pSaGap[ΔVI] | PR01 | This work |
| L2ALS38 | pSaGap[ΔVI] | PR01-02 | This work |
| L2ALS42 | pSaGap[ΔV∆VI] | PR01 | This work |
| L2ALS43 | pSaGap[ΔV∆VI] | PR01-02 | This work |
| L2ALS45 | pSaGap | PR01 | This work |
| L2ALS46 | pSaGap | PR01-02 | This work |
| L2ALS54 | pSaGap[ΔIV] | PR01 | This work |
| L2ALS59 | pSaGap[ΔIV] | PR01-02 | This work |
| L2ALS74 | pSaGap[InvStem] | PR01 | This work |
| L2ALS77 | pSaGap[InvStem] | PR01-02 | This work |
| L2ALS80 | pSaGap[ΔI∆VI] | PR01 | This work |
| L2ALS83 | pSaGap[ΔI∆VI] | PR01-02 | This work |
| L2ALS101 | pSaGap[∆128] | PR01 | This work |
| L2ALS103 | pSaGap[∆128] | PR01-02 | This work |
| L2ALS105 | pSaGap[ΔI∆II] | PR01 | This work |
| L2ALS107 | pSaGap[ΔI∆II] | PR01-02 | This work |
| L2ALS109 | pSaGap[ΔIΔII+G] | PR01 | This work |
| L2ALS111 | pSaGap[ΔIΔII+G] | PR01-02 | This work |
| L2ALS113 | pBsCggshort | PR01 | This work |
| L2ALS115 | pBsCggshort | PR01-02 | This work |
| L2ALS117 | pSaGap[A264U] | PR01 | This work |
| L2ALS119 | pSaGap[A264U] | PR01-02 | This work |
| L2ALS121 | pSaGap[U280A] | PR01 | This work |
| L2ALS123 | pSaGap[U280A] | PR01-02 | This work |
| L2ALS125 | pSaGap[ΔIV::CggHP] | PR01 | This work |
| L2ALS127 | pSaGap[ΔIV::CggHP] | PR01-02 | This work |
| L2ALS129 | pSaGap[G268C] | PR01 | This work |
| L2ALS131 | pSaGap[G268C] | PR01-02 | This work |
| L2ALS147 | pSaGln | PR01 | This work |
| L2ALS149 | pSaGln | PR01-02 | This work |
| L2ALS153 | pSaGln | L2ALS01 | This work |
| L2ALS155 | pBsGln | PR01 | This work |
| L2ALS157 | pBsGln | PR01-02 | This work |
| L2ALS161 | pBsGln | L2ALS01 | This work |
| L2ALS163 | pSaGap[G268C,C276G] | PR01 | This work |
| L2ALS165 | pSaGap[G268C,C276G] | PR01-02 | This work |
| L2ALS167 | pSaGap[A264U,U280A] | PR01 | This work |
| L2ALS168 | pSaGap[A264U,U280A] | PR01-02 | This work |
| L2ALS169 | pSaGap[C276G] | PR01 | This work |
| L2ALS170 | pSaGap[C276G] | PR01-02 | This work |
| L2ALS171 | pSaGap[InvStemGC] | PR01 | This work |
| L2ALS172 | pSaGap[InvStemGC] | PR01-02 | This work |
| L2ALS181 | pSaGap[IIIrandom] | PR01 | This work |
| L2ALS212 | pSaGln[GtoC] | PR01 | This work |
| L2ALS214 | pSaGln[GtoC] | PR01-02 | This work |
| L2ALS216 | pSaGln[GtoC,CtoG] | PR01 | This work |
| L2ALS218 | pSaGln[GtoC,CtoG] | PR01-02 | This work |
| L2ALS236 | pBsCgg[GtoC] | PR01 | This work |
| L2ALS238 | pBsCgg[GtoC] | PR01-02 | This work |
| L2ALS242 | pBsCgg[GtoC,CtoG] | PR01 | This work |
| L2ALS244 | pBsCgg[GtoC,CtoG] | PR01-02 | This work |
| L2ALS248 | pSaGap[ΔV] | PR01 | This work |
| L2ALS250 | pSaGap[ΔV] | PR01-02 | This work |
| L2ALS262 | pSaGap[NoStart] | PR01 | This work |
| L2ALS263 | pSaGap[NoStart] | PR01-02 | This work |
| L2ALS264 | pSaGap[ΔII+G] | PR01 | This work |
| L2ALS265 | pSaGap[ΔII+G] | PR01-02 | This work |
| L2ALS266 | p*fliM*::II-V | PR01 | This work |
| L2ALS267 | p*fliM*::II-V | PR01-02 | This work |
| L2ALS268 | p*fliM* | PR01 | This work |
| L2ALS269 | p*fliM* | PR01-02 | This work |
| **Strain**  **name** | **Description** | **Parent strain** | **Reference** |
| *Bacillus subtilis* strains | | | |
| SSB1002 | Wild-type |  |  |
| CCB441 | *rny::spc* | SSB1002 |  |
| CCB1111 | *amyE::pHM2-cvfA rny:spc* | SSB1002 |  |
| CCB1112 | *amyE::pHM2-rny rny::spc* | SSB1002 |  |
| *E. coli* strains | | | |
| DH5α |  |  | Lab strain |
| Stellar |  |  | Takara |

## References

1. Redder P, Linder P (2012) New range of vectors with a stringent 5-fluoroorotic acid-based counterselection system for generating mutants by allelic replacement in Staphylococcus aureus. Appl Environ Microbiol 78:3846–3854. https://doi.org/10.1128/AEM.00202-12
