# Supplementary material for: Critical factors for precise and efficient RNA cleavage by RNase Y in Staphylococcus aureus
Source: PLoS Genet. 2024 Aug 1;20(8):e1011349. doi: 10.1371/journal.pgen.1011349 (PMC11321564; doi:10.1371/journal.pgen.1011349)
Supplement: S2 Table — (DOCX) [file pgen.1011349.s002.docx]

### S2 Table. List of oligos:

| **Oligo number or name** | **Sequence in 5' to 3' direction (relevant restriction sites have been underlined)** | **Used for** |
| --- | --- | --- |
|  | **pSaGap construct** |  |
| 337 | TACGAGTCGACGATTAGCGAGTCAGTATAAG | pSaGap construct |
| 58 | TACTACGGCGCGCCTTAACCACCATCAACTACCTCT |  |
| 30 | ATTATTGGTACCGACCTTGAATCAAAAGACTT |  |
| 29 | TTATCTGGTACCGTCTTTCACTACTACCTCCTC |  |
|  | **pBsCgg construct** |  |
| 26 | AAAAAAGGTACCGCGGTAGCGGGCGGATCA | pBsCgg construct |
| 76 | CAACAAGGCGCGCCATCGTGGTTAGCCGCATCG |  |
| 53 | AAAAAAGGTACCAATATCGACCAGGTTCTGTTCC |  |
| 50 | AAAACTGTCGACTGGCGCTTTCTGTATAAGA |  |
| 431 | TAAATATCTCTCACTTATTTAAAGGAGG |  |
| 432 | ACTTCTTTGCGGCTCC |  |
| 556 | ATTATTCATGGGTTAATAACTTCTTTGCGGCTC | pBsCgg[GtoC] and pBsCgg[GtoC,CtoG] |
| 557 | CCCTCAATATAAATATCTCTCAC |  |
| 558 | GGGTCAATATAAATATCTCTCACTTATTTAAAG |  |
|  | **pSaGln construct** |  |
| 559 | GTCGACCGTTTTAGAAGTCGAAATCG | pSaGln construct |
| 560 | TTAAATGCTCATAGTAACGTATTTGCCTTG |  |
| 561 | ACGTTACTATGAGCATTTAACAACAGATGAAC |  |
| 562 | GGCGCGCCTTAATCTGATTCTTCGATACGTAC |  |
| 592 | TGCCTGCAGGTCGACCGTTTTAGAAGTCGAAATCGCA |  |
| 593 | TAGAATAGGCGCGCCTTAATCTGATTCTTCGATACGTACGAA |  |
| 700 | GGAGCATTTATTGGCAAAGTTTCTCC | pSaGln[GtoC] and pSaGln[GtoC,CtoG] |
| 701 | TGATTTATGGGCATTTATTAAATAAAATTTGGAGGATT |  |
| 702 | TGATTTATCCCGATTTATTAAATAAAATTTG |  |
|  | **pBsGln construct** |  |
| 565 | ATGTTTCATTGGATAATATCGAATTTGTCTTGCT | pBsGln construct |
| 566 | TTCGATATTATCCAATGAAACATGATCTGTC |  |
| 580 | TTTGTCGACTTTCTCTGGATTTG |  |
| 581 | TTTGGCGCGCCTTACTCTTC |  |
| 582 | TGCCTGCAGGTCGACTTTCTCTGGATTTGATGTTAAGAATCC |  |
| 583 | TAGAATAGGCGCGCCTTACTCTTCGATACGAACGAATCC |  |
|  | **pSaGap mutations** |  |
| 333 | TCGTATAGGCGCGCCTTAACCACC | pSaGap[∆III] |
| 334 | TCGTAGGTACCGTCTTTCACTACTACC | pSaGap[∆I], pSaGap[∆I∆II], pSaGap[∆I∆II+G] |
| 337 | TACGAGTCGACGATTAGCGAGTCAGTATAAG | pSaGap[∆III] |
| 338 | TGAATAAGTAAAAAGTTTAATACTTTTTAAATATC | pSaGap[∆III] |
| 339 | AAAGTATTAAACTTTTTACTTATTCAAGTATTATCTTTGCTG |  |
| 342 | TACGAGGTACCATACTTGAATAAGAGATAAAAAG | pSaGap[∆I] |
| 343 | AATCAGAATTCGTTAAGGCGCGCCTATTCTAAATGC | pSaGap[∆VI], pSaGap[∆V∆I] |
| 344 | TACTGAATTCTACCAAAACCATTAATTGCT | pSaGap[∆VI] |
| 345 | TACTGGAATTCTTAAAAAGTATTAAACTTTTTATCTCTTA | pSaGap[∆V∆I] |
| 421 | AATATCATTTTAAAGGAGGCCATTATA | pSaGap[∆IV] |
| 424 | TCTCTTATTCAAGTATTATCTTTGCTG |  |
| 423 | AATTGAAAAATAATATCATTTTAAAGGAGGCC | pSaGap[InvStem] |
| 424 | AATGAAAAATTCTCTTATTCAAGTATTATCTTTGCTG |  |
| 478 | TACGAGGTACCAATACTTGAATAAGAGATAAAAAG | pSaGap[∆128] |
| 29 | TTATCTGGTACCGTCTTTCACTACTACCTCCTC |  |
| 479 | TTTGGTACCAGATAAAAAGTTTAATACTTTTTAAATATCAT | pSaGap[∆I∆II] |
| 480 | TTTGGTACCGAGATAAAAAGTTTAATACTTTTTAAATATC | pSaGap[∆I∆II+G] |
| 513 | ATATCTCTTATTCAAGTATTATCTTTGC | pSaGap[A264U] |
| 514 | AAAGTTTAATACTTTTTAAATATCATTTTAAAG |  |
| 515 | TAAAGTATTAAACTTTTTATCTCTTATTC | pSaGap[U280A] |
| 516 | TAAATATCATTTTAAAGGAGGCC |  |
| 517 | TAATCCCTCAATATATATCATTTTAAAGGAGGCC | pSaGap[cggRSL] |
| 518 | TTCATCCCTTAATAACTCTTATTCAAGTATTATCTTTGC |  |
| 553 | GTTTTTATCTCTTATTCAAGTATTATCT | pSaGap[G268C] |
| 554 | TTTAATACTTTTTAAATATCATTTTAAAGG |  |
| 563 | TAAAGTATTAAACTTTATATCTCTTATTCAAG | pSaGap[A264U,U280A] |
| 634 | NNNTAAAAAGTTTAATACTTTTTAAATATCATTTT | pSaGap[IIINNN] (variant) |
| 636 | CTTATTCAAGTATTATCTTTGCTG |  |
| 640 | TAAAGTATTAAACTTTATATCTCTTATTCAAG | pSaGap[A264U,U280A] |
| 641 | AATTCAAAAATAATATCATTTTAAAGGAGGC | pSaGap[InvStemGC] |
| 642 | AATCAAAAATTCTCTTATTCAAGTATTATCTTT |  |
| 643 | TTTAATAGTTTTTAAATATCATTTTAAAGGAGGC | pSaGap[C276G] |
| 644 | CTTTTTATCTCTTATTCAAGTATTATCTT |  |
| 706 | TATCTTTGCTGCGGCTTC | pSaGap[∆II+G] |
| 707 | GAGATAAAAAGTTTAATACTTTTTAAATATC |  |
| 708 | ATATTTAAAAAGTATTAAACTTTTTATCTCTTATT | pSaGap[∆V] |
| 709 | GGTCGTTTAGCATTCAGAAG |  |
| 703 | TAAAAAGACGGTACCGACCTTG | pSaGap[NoStart] |
| 704 | TACTACCTCCTCCTTATATTTATAAATGTAAATAA |  |
| 799 | TAATTACTTTTTATCTCTTATTCAAGTATTATC | pSaGap[InvLoop] |
| 800 | ACTTTTTAAATATCATTTTAAAGGAGG |  |
| 925 | GCAGCAAAGATAGAGATAAAAAGTTTAATACTT | SecIImutant1 |
| 926 | CTCTATCTTTGCTGCTATCTTTGCTGCGGCTTC |  |
| 927 | TGGAGAAACTTTGAGATAAAAAGTTTAATACTT | SecIImutant2 |
| 928 | CTCAAAGTTTCTCCATATCTTTGCTGCGGCTTC |  |
| 935 | CTCGCCGAATAAGAGATAAAAAGTTTAATACTT | SecIImutant3 |
| 936 | CTCTTATTCGGCGAGTATCTTTGCTGCGGCTTC |  |
| 937 | ATACTTCTCGCCGAGATAAAAAGTTTAATACTT | SecIImutant4 |
| 938 | CTCGGCGAGAAGTATTATCTTTGCTGCGGCTTC |  |
|  | **Cloning *fliM* *gap*** |  |
| 733 | GGCTGTTATACTTGAATAAGAGATAAAAAGTTT | *fliM* *gap* transcriptionnal fusion construct |
| 734 | TCAAGTATAACAGCCCCATACGAAAAT |  |
| 735 | TAGAATTCAACCTGCTGCGTCGTAGCC |  |
| 736 | GCAGGTTGAATTCTACCAAAACCATTAAT |  |
| 737 | GGCGCGCCTATTCTAAATG |  |
| 738 | TAGAATAGGCGCGCCAGCGTCCATCGCCGCCA |  |
| 739 | GTTAAAGTTTTACCAAGTATTCTTTCTCAAGCTG |  |
| 740 | TGGTAAAACTTTAACACAAGCATTAC |  |
| 794 | gataaaaagtttaatactttttaaatatcattt | pSaGap[IIIGGA] |
| 793 | ccttattcaagtattatctttgctgc |  |
| 796 | taaaaagtttaatactttttaaatatcattttaaa | Forward for pSaGap[IIIAUU], pSaGap[IIICUA] and pSaGap[IIICGA] |
| 795 | aatcttattcaagtattatctttgctgc | pSaGap[IIIAUU] |
| 797 | tagcttattcaagtattatctttgctg | pSaGap[IIICUA] |
| 798 | tcgcttattcaagtattatctttgctg | pSaGap[IIICGA] |
|  | **Northern blot** |  |
| 310 | TTAACTTCTGTGTTCGGCATGGGAACAGGTGTGACCTCC | Northern probe 5S |
| 1 | TCAAAATTATACATGTCAACGA | Northern probe P1 |
| 408 | GTGGTTGCCTTTTTAAGTCCCGCGTGGGAC | Northern probe P2 |
| CC058 (16S) | CAGCGTTCGTCCTGAGCCAG | Northern probe B. subtilis 16S |
| CC2437 (atpB) | CATAGTAGTGGGTTAAAGCAACAAC | Northern probe B. subtilis atpB |
| CC2438 (glnA) | TACACCGAACAATGGTTTTGGCATAA | Northern probe B. subtilis glnA |
|  | **EMOTE** |  |
| 298 | GGCATTCCTGCTGAACCGCTCTTCCGATCTTACATGTCAACGATAATACA | Reverse transcription in targeted EMOTE (This work) |
| BioRp8 | Biotin-dG-CGGCACCAACCGAGGVVVVVVVACAGA (RNA oligo) | EMOTE Ligation (Redder, 2018) |
| D6A | CTCTTTCCCTACACGACGCTCTTCCGATCTNTACACGGCACCAACCGAGG | Second strand PCR (Khemici et al., 2015) |
| D6B | CTCTTTCCCTACACGACGCTCTTCCGATCTNGTATCGGCACCAACCGAGG |  |
| D6C | CTCTTTCCCTACACGACGCTCTTCCGATCTNCGTCCGGCACCAACCGAGG |  |
| D6D | CTCTTTCCCTACACGACGCTCTTCCGATCTNAAGTCGGCACCAACCGAGG |  |
| D6E | CTCTTTCCCTACACGACGCTCTTCCGATCTNACACCGGCACCAACCGAGG |  |
| D6F | CTCTTTCCCTACACGACGCTCTTCCGATCTNGGTACGGCACCAACCGAGG |  |
| D6H | CTCTTTCCCTACACGACGCTCTTCCGATCTNTCGGCGGCACCAACCGAGG |  |
| D6I | CTCTTTCCCTACACGACGCTCTTCCGATCTNCAAGCGGCACCAACCGAGG |  |
| D6J | CTCTTTCCCTACACGACGCTCTTCCGATCTNTTGACGGCACCAACCGAGG |  |
| D6K | CTCTTTCCCTACACGACGCTCTTCCGATCTNGCTGCGGCACCAACCGAGG |  |
| D6L | CTCTTTCCCTACACGACGCTCTTCCGATCTNCCGACGGCACCAACCGAGG |  |
| D6M | CTCTTTCCCTACACGACGCTCTTCCGATCTNCTCGCGGCACCAACCGAGG |  |
| D6N | CTCTTTCCCTACACGACGCTCTTCCGATCTNAGGACGGCACCAACCGAGG |  |
| D6O | CTCTTTCCCTACACGACGCTCTTCCGATCTNATTGCGGCACCAACCGAGG |  |
| D6P | CTCTTTCCCTACACGACGCTCTTCCGATCTNGACGCGGCACCAACCGAGG |  |
| D6Q | CTCTTTCCCTACACGACGCTCTTCCGATCTNTGTTCGGCACCAACCGAGG |  |
| A-PE-PCR10 | AATGATACGGCGACCACCGAGATCTACACTCTTTCCCTACACGACG |  |
| B-PE-PCR20 | CAAGCAGAAGACGGCATACGAGATCGGTCTCGGCATTCCTGCTGAACCGC |  |
|  | **DMS-MaPseq** |  |
| 835 | gcatctaatacgactcactatggcGTAAAGAGGTTAATTTTTGTCCCACGCGG | PCR to add T7 promoter to 540bp of gap operon. |
| 463 | cagtatttattatgcatttagaataggcgc |  |
| 868 | CTGGAGTTCAGACGTGTGCTCTTCCGATCTATGCTAAACGACCAATTCTA | RT-primer for DMS-MaPseq |
| 815 | CTCTTTCCCTACACGACGCTCTTCCGATCTNACAGTCACTGATGAAGCCGCAGC | Second strand synthesis, adding barcode “ACAG” |
| 816 | CTCTTTCCCTACACGACGCTCTTCCGATCTNTACCTCACTGATGAAGCCGCAGC | Second strand synthesis, adding barcode “TACC” |
| 817 | CTCTTTCCCTACACGACGCTCTTCCGATCTNCGTTTCACTGATGAAGCCGCAGC | Second strand synthesis, adding barcode “CGTT” |
| 818 | CTCTTTCCCTACACGACGCTCTTCCGATCTNTCGTTCACTGATGAAGCCGCAGC | Second strand synthesis, adding barcode “TCGT” |
| 233 | AATGATACGGCGACCACCGAGATCTACACTCTTTCCCTACACGACG | PCR amplification, forward primer |
| 255 | CAAGCAGAAGACGGCATACGAGATATTGGCGTGACTGGAGTTCAGACGTGTGC | PCR amplification, reverse primers |
| 259 | CAAGCAGAAGACGGCATACGAGATTGGTCAGTGACTGGAGTTCAGACGTGTGC |  |
| 260 | CAAGCAGAAGACGGCATACGAGATCACTGTGTGACTGGAGTTCAGACGTGTGC |  |
|  | **Sector III cleavage-efficiency quantification** |  |
| 762 | CTCTTTCCCTACACGACGCTCTTCCGATCTNTGGCAAAGATAATACTTGAATAAG | Plasmid quantification for the Sector III variant library |
| 763 | CTCTTTCCCTACACGACGCTCTTCCGATCTNGTGCAAAGATAATACTTGAATAAG |  |
| 764 | CTCTTTCCCTACACGACGCTCTTCCGATCTNACGCAAAGATAATACTTGAATAAG |  |
| 765 | CTCTTTCCCTACACGACGCTCTTCCGATCTNTCGCAAAGATAATACTTGAATAAG |  |
| 801 | CTCTTTCCCTACACGACGCTCTTCCGATCTNCAGCAAAGATAATACTTGAATAAG |  |
|  | **Generating *B. subtilis* mutants** |  |
| CC2228 | CATAGCAAGAGGAGGTGAAAGTGAATTTATTAAGCCTCCTAC | Cloning of SaY |
| CC2155 | GATGCTTAGCGCATCACTTTATTTCGCATATTCTACTGCTC |  |
| CC2150 | cactaaGAATTCTTGACAAGTATTTCCGAC | Cloning 5’end UTR of BsY |
| CC2229 | GTAGGAGGCTTAATAAATTCACTTTCACCTCCTCTTGCTATG |  |
| CC2154 | GAGCAGTAGAATATGCGAAATAAAGTGATGCGCTAAGCATC | Cloning 3’end UTR of BsY |
| CC2151 | CACTAAGTCGACTCTTCTTGAAAATTCCTTG |  |
