# Supplementary material for: Critical factors for precise and efficient RNA cleavage by RNase Y in Staphylococcus aureus
Source: PLoS Genet. 2024 Aug 1;20(8):e1011349. doi: 10.1371/journal.pgen.1011349 (PMC11321564; doi:10.1371/journal.pgen.1011349)
Supplement: S1 Fig — Adapted from Redder, 2018. (DOCX) [file pgen.1011349.s003.docx]

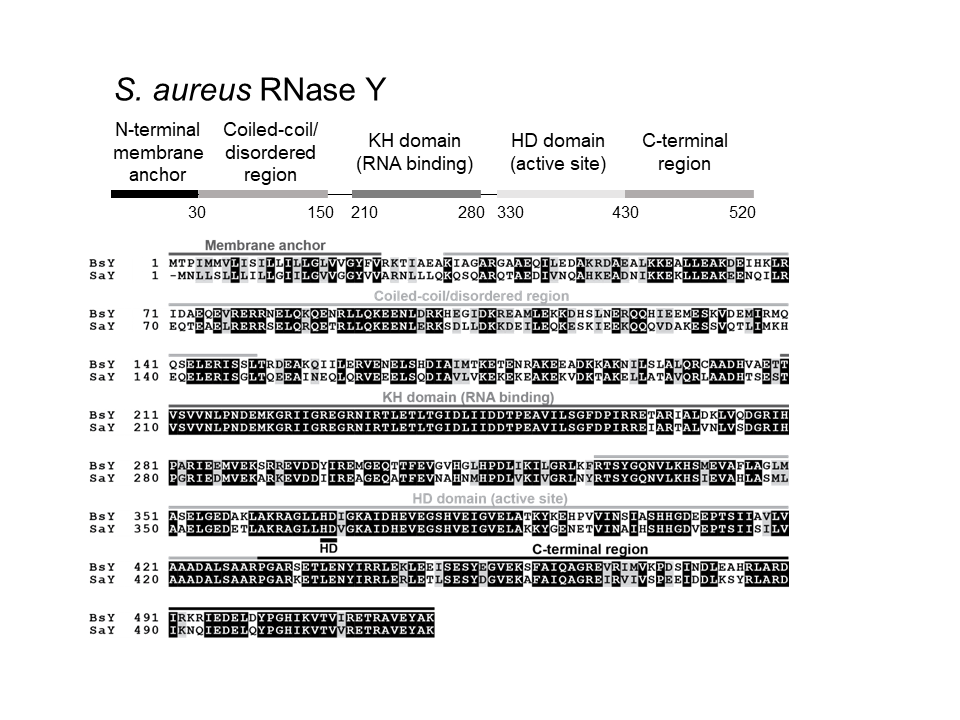


**S1 Fig. Alignment of *S. aureus* and *B. subtilis* RNase Y.** SaY: *S. aureus* RNase Y, BsY, *B. subtilis* RNase Y, HD: the two critical amino acids in the active site. Adapted from (1).

### Reference:

1. Redder P. Molecular and genetic interactions of the RNA degradation machineries in Firmicute bacteria. Wiley Interdiscip Rev RNA. 2018 Mar;9(2).
