# Supplementary material for: Critical factors for precise and efficient RNA cleavage by RNase Y in Staphylococcus aureus
Source: PLoS Genet. 2024 Aug 1;20(8):e1011349. doi: 10.1371/journal.pgen.1011349 (PMC11321564; doi:10.1371/journal.pgen.1011349)
Supplement: S2 Fig — A) The layout of the pSaGap construct, with the location of the upstream and downstream Northern blot probes (P2 and P1, respectively). B) Northern blot showing the appearance of two new bands when using the P2 (upstream) probe on total RNA from a PNPase deletion strain carrying pSaGap. Black and grey triangles indicate full-length transcript and cleavage products, respectively. This data is in agreement with a large-scale Streptococcus pyogenes study where upstream products of RNase Y cleavage are systematically degraded by PNPase [20]. (DOCX) [file pgen.1011349.s004.docx]

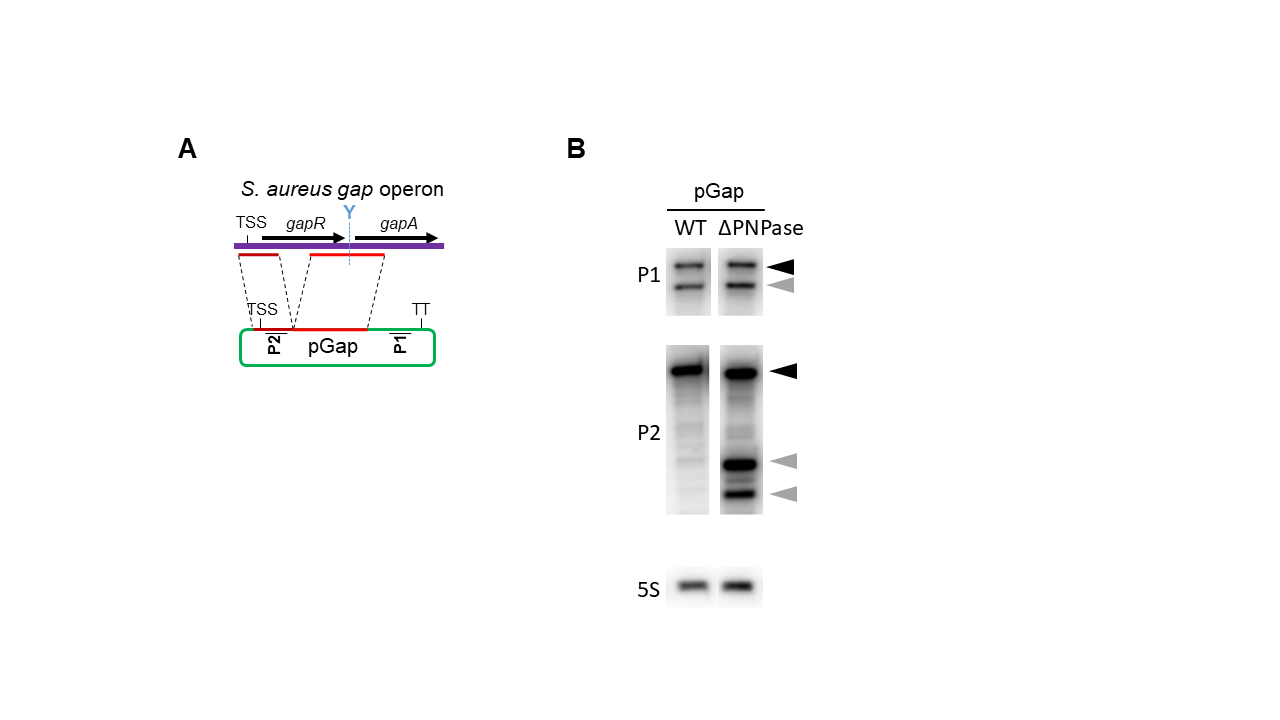


**S2 Fig. PNPase degrades the upstream cleavage fragment of the pSaGap transcript.**

A) The layout of the pSaGap construct, with the location of the upstream and downstream Northern blot probes (P2 and P1, respectively).

B) Northern blot showing the appearance of two new bands when using the P2 (upstream) probe on total RNA from a PNPase deletion strain carrying pSaGap. Black and grey triangles indicate full-length transcript and cleavage products, respectively.

This data is in agreement with a large-scale *Streptococcus pyogenes* study where upstream products of RNase Y cleavage are systematically degraded by PNPase (1).

## Reference

1. Broglia L, Lécrivain A-L, Renault TT, et al (2020) An RNA-seq based comparative approach reveals the transcriptome-wide interplay between 3’-to-5’ exoRNases and RNase Y. Nat Commun 11:1587. https://doi.org/10.1038/s41467-020-15387-6
