# Supplementary material for: Critical factors for precise and efficient RNA cleavage by RNase Y in Staphylococcus aureus
Source: PLoS Genet. 2024 Aug 1;20(8):e1011349. doi: 10.1371/journal.pgen.1011349 (PMC11321564; doi:10.1371/journal.pgen.1011349)
Supplement: S3 Fig — The EMOTE data is presented as proportions of RNA molecules with a given 5’ end on the Y-axis (number of reads detected at a specific position divided by the total number of reads detected within the chosen window). Note that the number of detected molecules with 5’ends within the shown windows is much larger in the WT strain than in the ΔY strain, since RNase Y does not cleave the RNAs in the ΔY strain. The ΔY data is therefore based on a very low number of detected molecules and corresponds to background noise. The random nature of this noise can sometimes lead to a tall column for a position, since the EMOTE data is presented as proportions, with sum of the columns set to 1 (this is for example the case for the 6th position in the ΔY pSaGln data on panel A, where no cleavage is observed in the Northern blot in Fig 1C). The native RNase Y cleavage position is indicated with blue dotted lines. Green dotted lines indicate shifted cleavage positions where relevant. A) Corresponds to Fig 1. B) Corresponds to Fig 4C. C) Corresponds to Fig 4D. D) Corresponds to Fig 6. E) Corresponds to Fig 8. (DOCX) [file pgen.1011349.s005.docx]

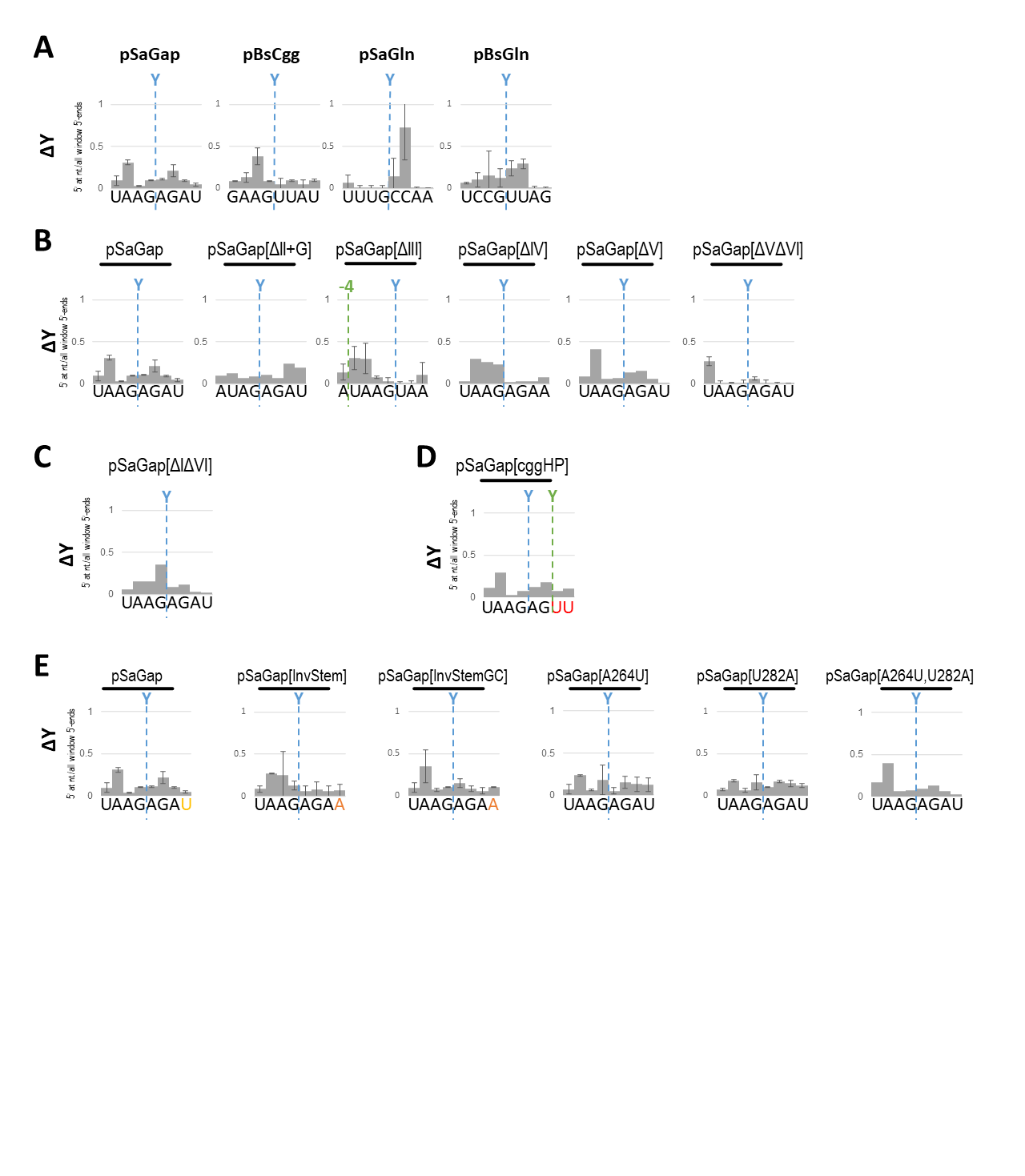


**S3 Fig. EMOTE data from the ΔY strain corresponding to Figs 1, 4, 6 and 8.**

The EMOTE data is presented as proportions of RNA molecules with a given 5’ end on the Y-axis (number of reads detected at a specific position divided by the total number of reads detected within the chosen window).

Note that the number of detected molecules with 5’ends within the shown windows is much larger in the WT strain than in the ΔY strain, since RNase Y does not cleave the RNAs in the ΔY strain. The ΔY data is therefore based on a very low number of detected molecules and corresponds to background noise. The random nature of this noise can sometimes lead to a tall column for a position, since the EMOTE data is presented as proportions, with sum of the columns set to 1 (this is for example the case for the 6^th^ position in the ΔY pSaGln data on panel A, where no cleavage is observed in the Northern blot in Fig 1C).

The native RNase Y cleavage position is indicated with blue dotted lines. Green dotted lines indicate shifted cleavage positions where relevant. A) Corresponds to Fig 1. B) Corresponds to Fig 4C. C) Corresponds to Fig 4D. D) Corresponds to Fig 6. E) Corresponds to Fig 8.
