# Supplementary material for: Critical factors for precise and efficient RNA cleavage by RNase Y in Staphylococcus aureus
Source: PLoS Genet. 2024 Aug 1;20(8):e1011349. doi: 10.1371/journal.pgen.1011349 (PMC11321564; doi:10.1371/journal.pgen.1011349)
Supplement: S4 Fig — A) Northern blot showing RNase Y dependent cleavage in pSaGap with a frame-shift (pSaGap[Δ128]) and with the start-codon mutated (pSaGap[NoStart]). B) EMOTE data confirming that the frame-shift in pSaGap[Δ128] has not shifted the RNase Y cleavage position. (DOCX) [file pgen.1011349.s006.docx]

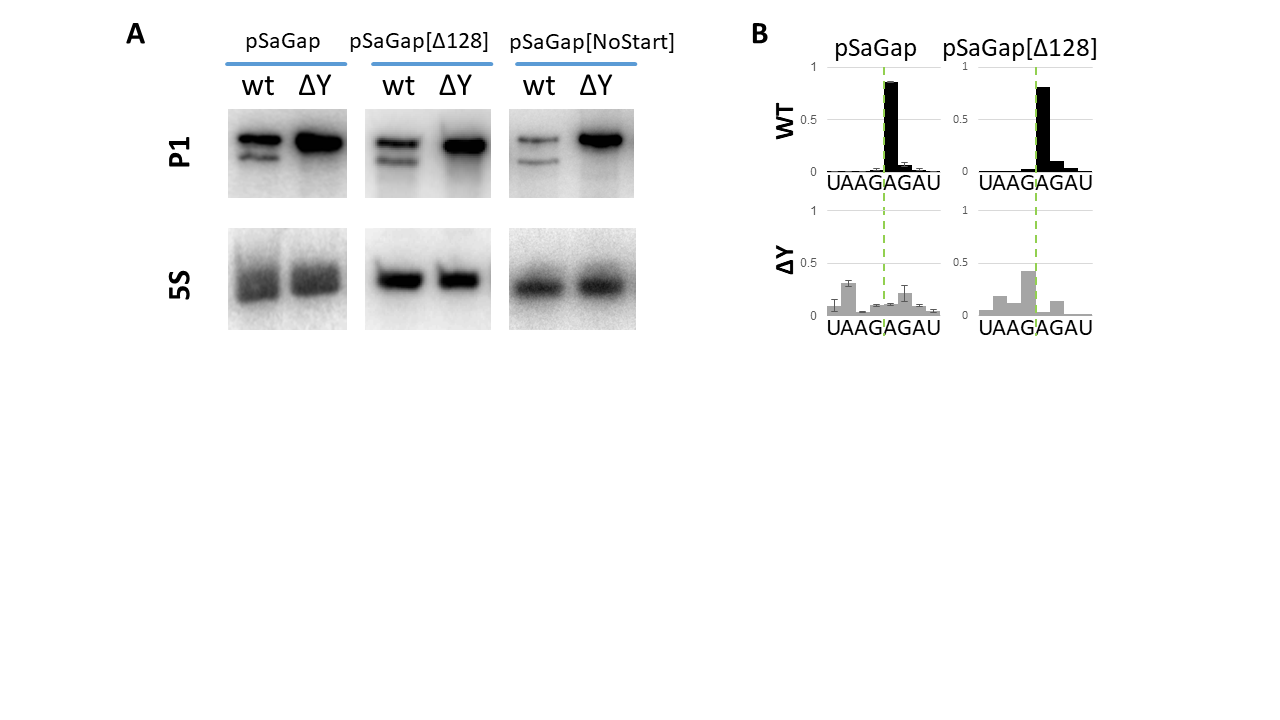


**S4 Fig. Translation does not influence RNase Y cleavage.** A) Northern blot showing RNase Y dependent cleavage in pSaGap with a frame-shift (pSaGap[Δ128]) and with the start-codon mutated (pSaGap[NoStart]). B) EMOTE data confirming that the frame-shift in pSaGap[Δ128] has not shifted the RNase Y cleavage position.
