# Supplementary material for: Critical factors for precise and efficient RNA cleavage by RNase Y in Staphylococcus aureus
Source: PLoS Genet. 2024 Aug 1;20(8):e1011349. doi: 10.1371/journal.pgen.1011349 (PMC11321564; doi:10.1371/journal.pgen.1011349)
Supplement: S5 Fig — A) Northern blot for the deletions of sectors I and II of pSaGap, with or without the G upstream of the cleavage position. B) Putative secondary structure that can form if both sectors I and II of pSaGap are deleted. Native RNase Y cleavage positions are indicated with blue dotted lines. Sector III is highlighted in green. C) The sequences of the wild-type pSaGap sector II and the four mutated versions. The G immediately upstream of the cleavage site has been underlined, and the nucleotides that differ from the wild-type sequence are shown in red. D) Northern blot showing that while the transcript from pSaGap[ΔII+G] is not cleaved, the transcripts from all four mutant variants are cleaved. The strain background is shown below each lane. The full-length and cleaved transcripts are indicated by the black and grey arrowheads, respectively. E) Northern blot showing cleavage of pBsCgg and BsCgg[Δ12] (where 12 nucleotides immediate upstream of the conserved G have been deleted). F) Northern blot showing cleavage of pBsGln and BsGln[Δ12] (where 12 nucleotides immediate upstream of the conserved G have been deleted). A weak band corresponding to the cleaved product has been marked with an asterisk. (DOCX) [file pgen.1011349.s007.docx]

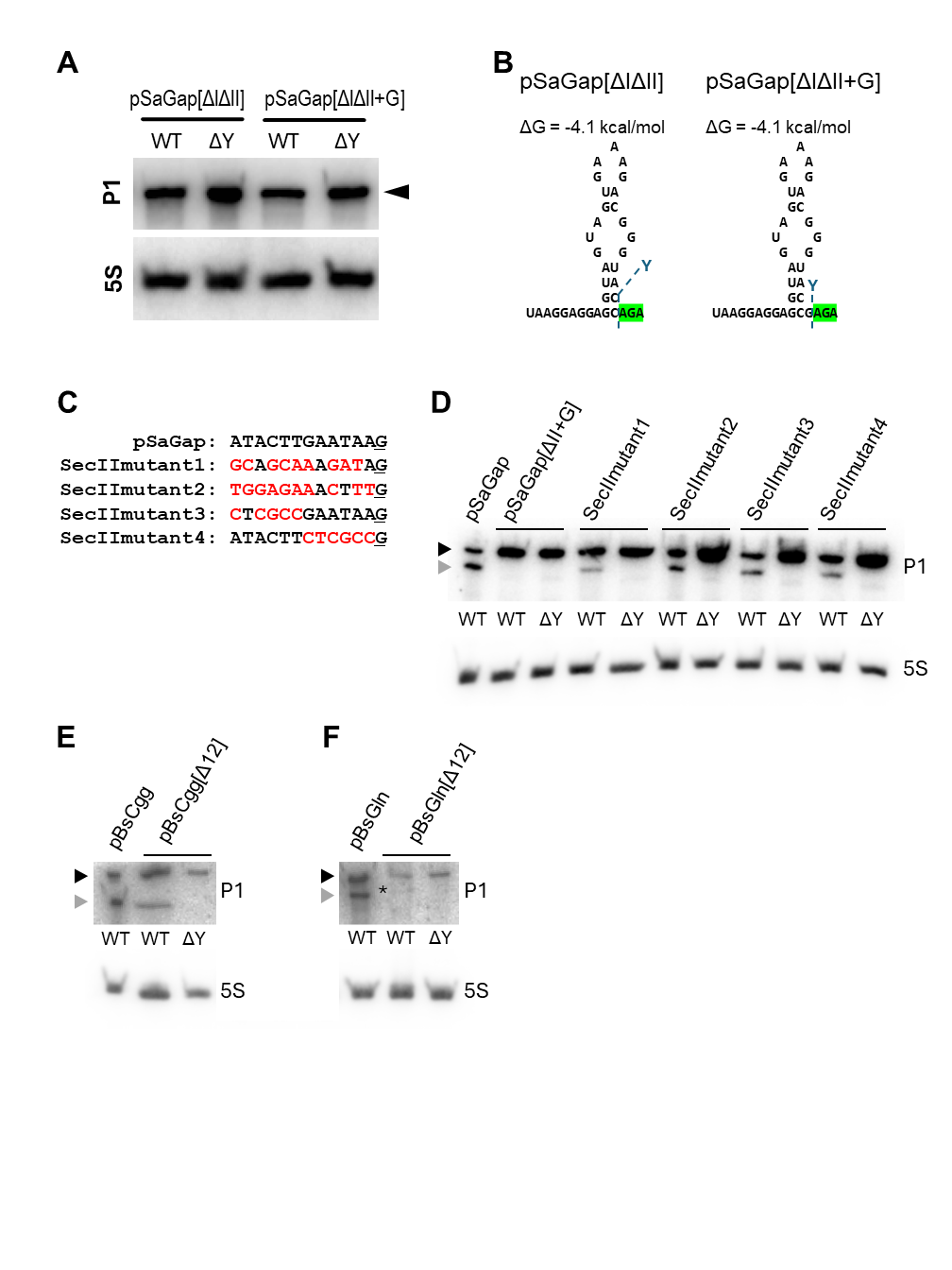


**S5 Fig. Mutations upstream of the RNase Y cleavage sites.**

A) Northern blot for the deletions of sectors I and II of pSaGap, with or without the G upstream of the cleavage position.

B) Putative secondary structure that can form if both sectors I and II of pSaGap are deleted. Native RNase Y cleavage positions are indicated with blue dotted lines. Sector III is highlighted in green.

C) The sequences of the wild-type pSaGap sector II and the four mutated versions. The G immediately upstream of the cleavage site has been underlined, and the nucleotides that differ from the wild-type sequence are shown in red.

D) Northern blot showing that while the transcript from pSaGap[ΔII+G] is not cleaved, the transcripts from all four mutant variants are cleaved. The strain background is shown below each lane. The full-length and cleaved transcripts are indicated by the black and grey arrowheads, respectively.

E) Northern blot showing cleavage of pBsCgg and BsCgg[Δ12] (where 12 nucleotides immediate upstream of the conserved G have been deleted).

F) Northern blot showing cleavage of pBsGln and BsGln[Δ12] (where 12 nucleotides immediate upstream of the conserved G have been deleted). A weak band corresponding to the cleaved product has been marked with an asterisk.
