# Supplementary material for: Critical factors for precise and efficient RNA cleavage by RNase Y in Staphylococcus aureus
Source: PLoS Genet. 2024 Aug 1;20(8):e1011349. doi: 10.1371/journal.pgen.1011349 (PMC11321564; doi:10.1371/journal.pgen.1011349)
Supplement: S6 Fig — A) The putative hairpin immediately downstream of the RNase Y cleavage in pBsGln. B) The sequence of the putative InvLoop hairpin where the loop has been modified so that U becomes A and A becomes U (mutated nucleotides shown in red). C) Northern blot showing the cleavage of the pSaGap[InvLoop] transcript. (DOCX) [file pgen.1011349.s008.docx]

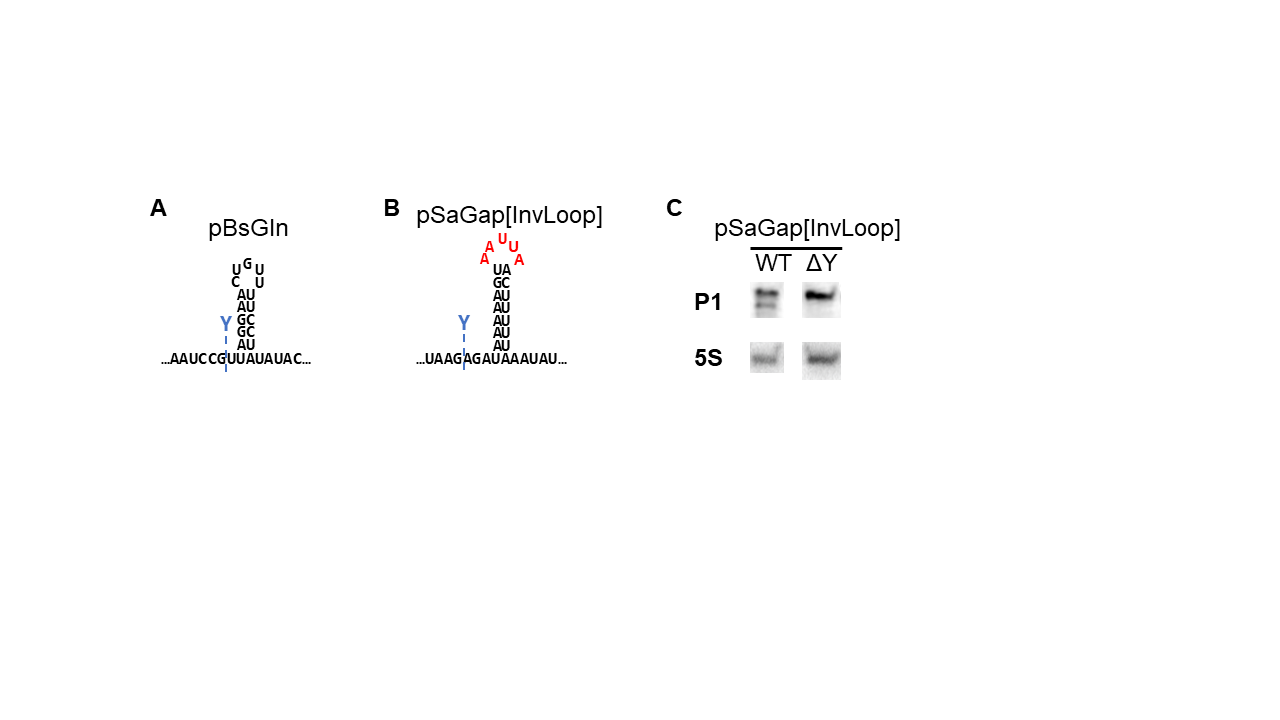


S6 **Fig. Putative hairpin in pBsGln and inversion of the putative hairpin loop in Sector IV of pSaGap.**

A) The putative hairpin immediately downstream of the RNase Y cleavage in pBsGln.

B) The sequence of the putative InvLoop hairpin where the loop has been modified so that U becomes A and A becomes U (mutated nucleotides shown in red).

C) Northern blot showing the cleavage of the pSaGap[InvLoop] transcript.
