# Supplementary material for: Critical factors for precise and efficient RNA cleavage by RNase Y in Staphylococcus aureus
Source: PLoS Genet. 2024 Aug 1;20(8):e1011349. doi: 10.1371/journal.pgen.1011349 (PMC11321564; doi:10.1371/journal.pgen.1011349)
Supplement: S7 Fig — Note that the EMOTE data is presented as proportions of RNA molecules with a given 5’ end on the Y-axis (number of reads detected at a specific position divided by the total number of reads detected within the chosen window), and that the number of detected molecules is much larger in the WT strain than in the ΔY strain. (DOCX) [file pgen.1011349.s009.docx]

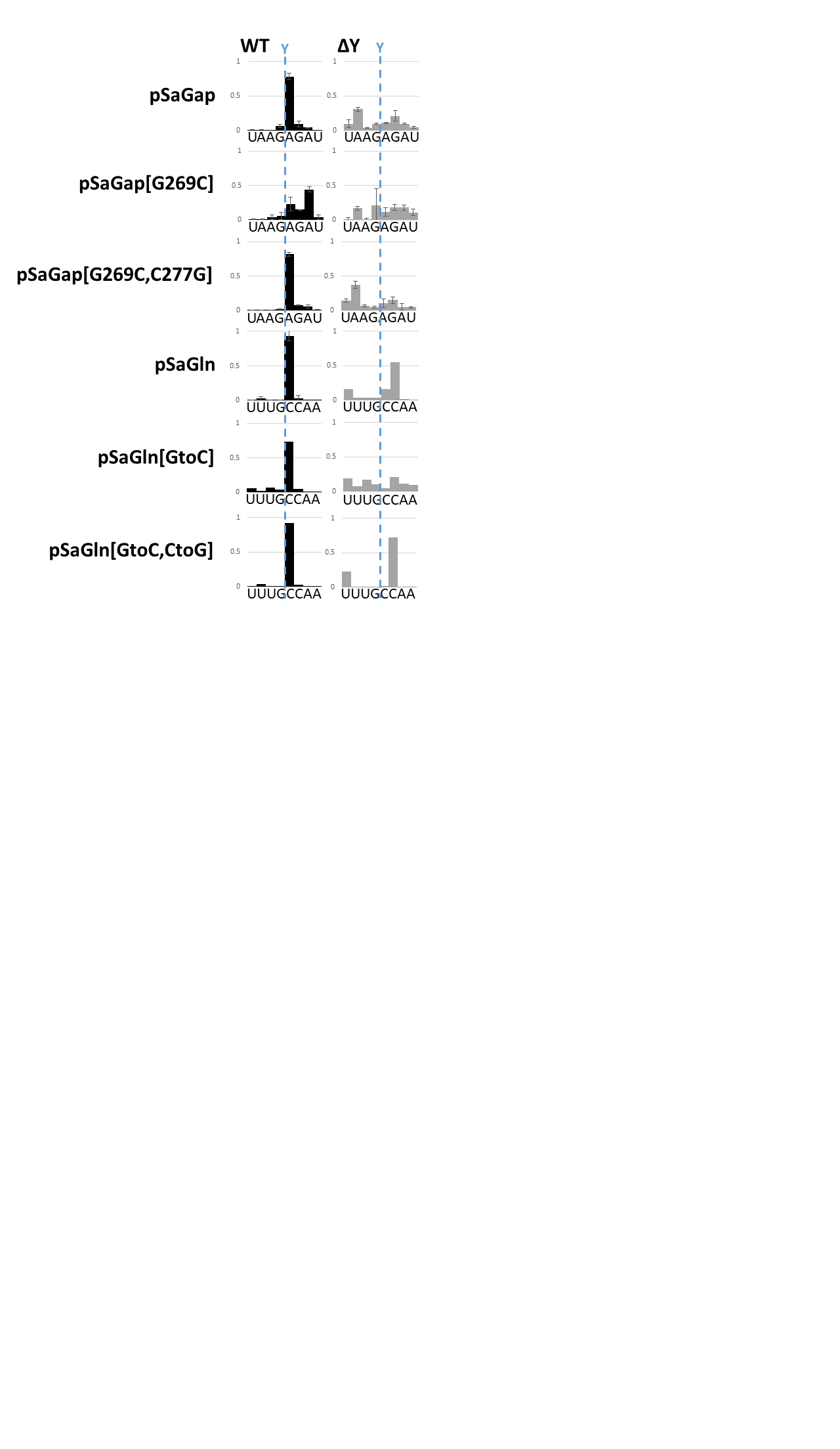


**S7 Fig.** **EMOTE data for hairpins with mutated G-C base-pairs, corresponding to Fig 7A and 7C in the main text.** Note that the EMOTE data is presented as proportions of RNA molecules with a given 5’ end on the Y-axis (number of reads detected at a specific position divided by the total number of reads detected within the chosen window), and that the number of detected molecules is much larger in the WT strain than in the ΔY strain.
