# Supplementary material for: Critical factors for precise and efficient RNA cleavage by RNase Y in Staphylococcus aureus
Source: PLoS Genet. 2024 Aug 1;20(8):e1011349. doi: 10.1371/journal.pgen.1011349 (PMC11321564; doi:10.1371/journal.pgen.1011349)
Supplement: S8 Fig — A) Wild-type (AGA) Sa-gapR hairpin, hairpin with a single base-pair extension (NVU) and hairpin with two base-pairs extension (YUU). Y: Pyrimidine bases, V: A, C or G. Red nucleotides are varied and green U’s are the uridines that extend the putative hairpin stem by one or two base-pairs (NVU and YUU, respectively). B) EMOTE data from NVU trinucleotide combinations that can potentially extend the stem by a single base-pair. The cleaved nucleotide sequence is shown below the graph and the native cleavage position is shown by a light blue dotted line. Red nucleotides are varied and the green U’s extend the putative hairpin stem by one base-pair. C) EMOTE data from YUU trinucleotide combinations that can potentially extend the stem by two base-pairs. The cleaved nucleotide sequence is shown below the graph and the native cleavage position is shown by a light blue dotted line. Red nucleotides are varied and the green U’s are the uridines that extend the putative hairpin stem by two base-pairs. (DOCX) [file pgen.1011349.s010.docx]

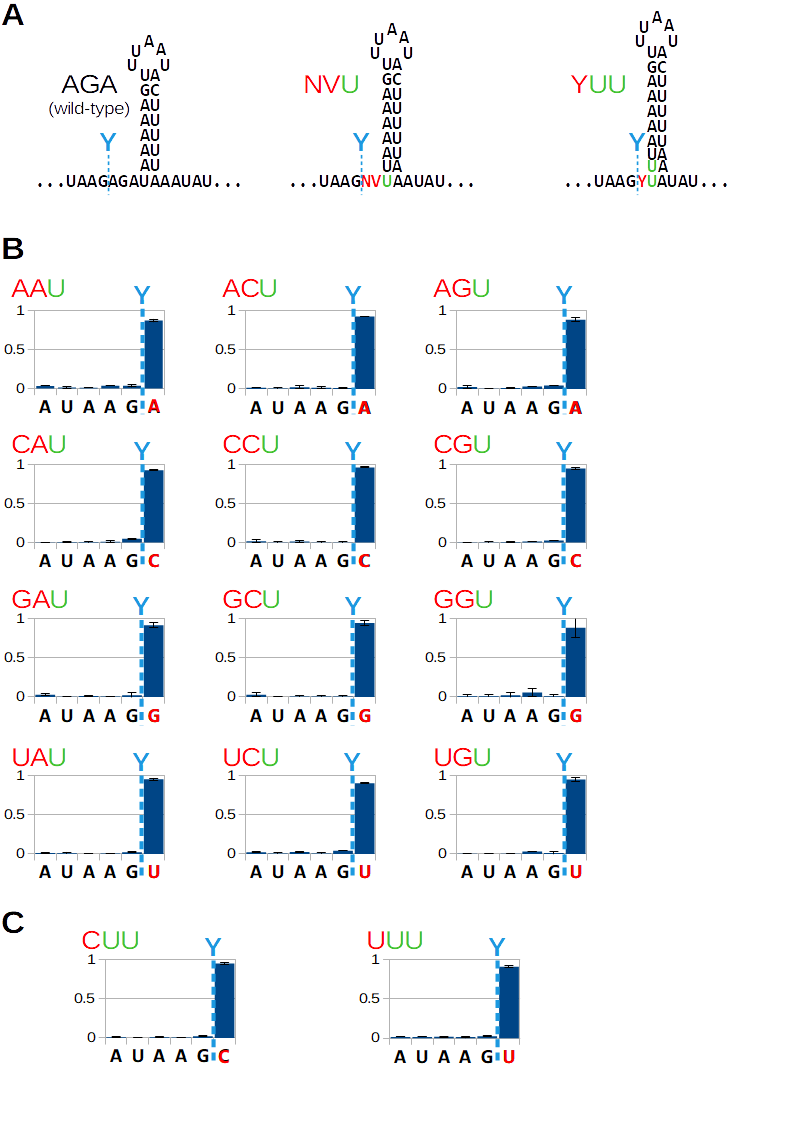


**S8 Fig. Extending the hairpin stem does not alter the cleavage position.**

A) Wild-type (AGA) *Sa-gapR* hairpin, hairpin with a single base-pair extension (NVU) and hairpin with two base-pairs extension (YUU). Y: Pyrimidine bases, V: A, C or G. Red nucleotides are varied and green U’s are the uridines that extend the putative hairpin stem by one or two base-pairs (NVU and YUU, respectively).

B) EMOTE data from NVU trinucleotide combinations that can potentially extend the stem by a single base-pair. The cleaved nucleotide sequence is shown below the graph and the native cleavage position is shown by a light blue dotted line. Red nucleotides are varied and the green U’s extend the putative hairpin stem by one base-pair.

C) EMOTE data from YUU trinucleotide combinations that can potentially extend the stem by two base-pairs. The cleaved nucleotide sequence is shown below the graph and the native cleavage position is shown by a light blue dotted line. Red nucleotides are varied and the green U’s are the uridines that extend the putative hairpin stem by two base-pairs.
