# Supplementary material for: Critical factors for precise and efficient RNA cleavage by RNase Y in Staphylococcus aureus
Source: PLoS Genet. 2024 Aug 1;20(8):e1011349. doi: 10.1371/journal.pgen.1011349 (PMC11321564; doi:10.1371/journal.pgen.1011349)
Supplement: S9 Fig — RNase Y cleavage sites are indicated with blue arrows. Structure prediction and free energy calculations were performed on the mFold.org server using default settings (Zuker 2003). A). The pfliM::II-V transcript. The RNase Y independent cleavage position is indicated in purple. B). pBsCgg transcript. C). pSaGln transcript. pBsGln transcript. (DOCX) [file pgen.1011349.s011.docx]

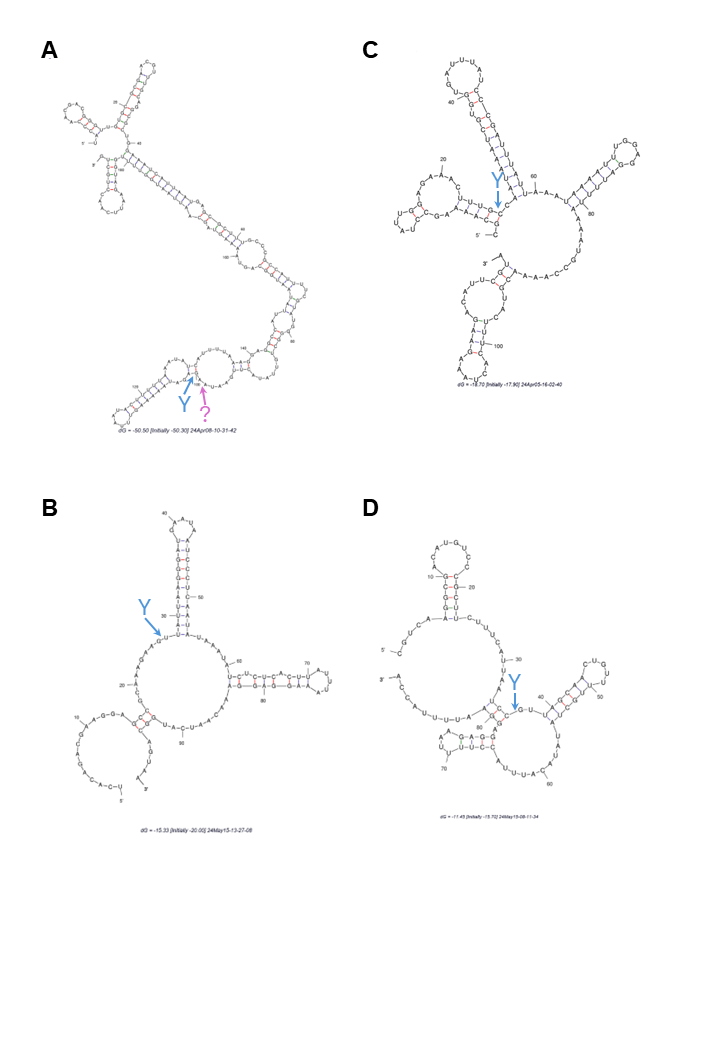


**S9 Fig. mFold predictions of secondary structures surrounding the RNase Y cleavage sites.** RNase Y cleavage sites are indicated with blue arrows. Structure prediction and free energy calculations were performed on the mFold.org server using default settings (1).

1. The pfliM::II-V transcript. The RNase Y independent cleavage position is indicated in purple.
2. pBsCgg transcript.
3. pSaGln transcript.
4. pBsGln transcript.

## References

1. Zuker M (2003) Mfold web server for nucleic acid folding and hybridization prediction. Nucleic Acids Res 31:3406–3415. https://doi.org/10.1093/nar/gkg595
