# Supplementary material for: Critical factors for precise and efficient RNA cleavage by RNase Y in Staphylococcus aureus
Source: PLoS Genet. 2024 Aug 1;20(8):e1011349. doi: 10.1371/journal.pgen.1011349 (PMC11321564; doi:10.1371/journal.pgen.1011349)
Supplement: Attachment — Submitted filename: Reply-to-reviewers_v6.docx [file pgen.1011349.s012.docx]

Dear Dr. Papenfort,

Thank you very much for agreeing to extend the 30 day deadline, the extension made it possible for us to perform additional experiments on SaGap sector II and its equivalents in other operons, as requested by the reviewers. Most of the additional experiments were carried out by three students in our team, whom we have added as co-authors for their involvement.

Please find below our point-for-point replies to the comments from the three reviewers.

The replies have been highlighted in red.

Sincerely,

Peter Redder

Reviewer #1: Rny is a membrane-localized RNase, which is thought to be responsible for the initiation of bulk mRNA turnover in firmicutes. However, Rny cleaves only a limited number of mRNAs. Cleavage may result in stabilisation of the processed mRNAs thereby playing an important role in regulation of co-transcribed genes. It is debated how Rny specificity is determined, and no clear processing site (besides a conserve G resiue) could be identified. Rny activity is likely determined by secondary structure recognition determinants, downstream of the cleavage site.

Here the authors analysed Rny dependent operon processing of two homologous transcripts present in B. subtilis and S. aureus. They could show that Rny dependent processing is highly similar in both species. Through thorough mutant analyses they could show that cleavage is dependent on secondary structure in close vicinity to the processing site. The results were gained through Northern blot analysis and startsite predictions using EMOTE. This is a well-written manuscript with well suited experiments.

Results are mainly based on the SaGap operon and northern blot/EMOTE analyses. It would be more convincing if the main results could be reproduced in at least one more operon.

Reply: We agree that detailed data for additional operons would be helpful. While we have not reproduced all our experiments from the SaGap transcript in other transcripts, we have presented experiments that show the importance of the downstream hairpin in two additional transcripts (SaGln and BsCgg). We drastically reduce cleavage efficiency when we disrupt the base-pairing in the downstream hairpins of the two transcripts, and when we re-constitute the base-pairing in an inverted form, we re-gain cleavage (Figure 7), thus confirming that one of our major findings for SaGap is also applicable to other transcripts (but perhaps not all, since not all cleaved transcripts appear to have a hairpin downstream of their cleavage position).

We would have liked to perform DMS-MaPseq on more of our transcript, but unfortunately the special thermostable Group II Reverse Transcriptase (TGIRTIII from Ingex) we were using for DMS-MapSeq was discontinued in 2023, and is no longer available. Enzymes with similar properties are now commercially available, such as Induro reverse transcriptase from NEB and Marathon from Yale university. However, it requires a new and complete optimization of RT conditions and comparison of efficacy with robust statistics and this is not doable within the time given for revisions of this manuscript.

Additionally, we have performed additional experiments which determine that the role of the upstream sequence (sector II) appears to be different in other transcripts (please also see the reply to the comment about Line 683). We have added the following paragraph to the manuscript to describe our findings:

“The role of sector II was further explored by generating four mutated versions of sector II, all of which retained the G residue: SecIImutant1, where the 12 nucleotides from the hairpin of sector I replaced the 12 nucleotides of sector II; SecIImutant2, where sector II was replaced by the 12 equivalent nucleotides from the SaGln transcript; SecIImutant3, where the first half of sector II was mutated and SecIImutant4, where the second half of sector II was mutated (S5C Fig). All four of these mutant transcripts were cleaved in an RNase Y-dependent manner (S5D Fig), suggesting that sector II must be present for cleavage to occur, but that its sequence is of little to no importance. To determine whether a similar deletion would have the same effect on other transcripts, we deleted the 12 nucleotides upstream of the conserved G residue of pBsCgg and pBsGln (generating pBsCgg[Δ12] and pBsGln[Δ12], respectively), and observed that both of the mutant transcripts were cleaved by RNase Y, although the efficiency of cleavage appears to be lower in pBsGln[Δ12] (S5E Fig and S5F Fig).”

Specific comments

Line 57: reference Meinken et is not appropriate for the statement

Reply: Corrected.

Line 77: Add reference for the statement on the membrane anchor

Reply: The reference Khemici et al 2015 has been added.

Line 90: The interaction of RNaseY with auxillary proteins (YlbF, YmcA, and

YaaT (the Y-complex) should be introduced here.

Reply: We have added an introduction to the Y-complex.

Line 225: add a reference to the Fig.

Reply: Figure 2 is referenced in the paragraph entitled: ” S. aureus RNase Y is functional in B. subtilis.”

Figure 4: The meaning of the asterisk in Fig 4C is not clear. Why are the results not shown.

Reply: The asterisk refers to Figure S5B, where the EMOTE data from the double-deletion of sectors I and VI (vector pSaGap[ΔIΔIV]) are presented. We did not generate EMOTE data for the sector I and sector VI deletions individually (since they did not appear to impact cleavage).

We agree with reviewer 1 that this could be made clearer for the reader, so we have now moved the EMOTE data from Figure S5B to Figure 4D to have the EMOTE data in the same figure.

Line 374: The analysis of the SaGap operon is very extensive and conclusive. It would be very helpful to analyse the other operons for similar structures (e.g. the loop of sector IV)? The validation of at least one of these structures would be very helpful .

Reply: We agree that it is important to expand the conclusions from SaGap to other transcripts, and we therefore analysed the predicted hairpins downstream of the cleavage sites in the BsCgg and SaGln transcripts by mutational disruption of the G-C pairs in the hairpin stems, which led to almost complete abolishment of cleavage. This was followed by mutations that repaired the putative duplex formation, but in inverted orientation. These “repaired” hairpins allowed cleavage to occur, showing that the secondary structure is important for cleaving the BsCgg and SaGln transcripts as well (Figure 7). To better highlight this analysis for the reader, we have the following sentence in the discussion: ”Furthermore, our mutational analyses of the putative hairpins in the pSaGap, pBsCgg and pSaGln transcripts revealed that the putative G-C base-pairs are crucial for cleavage efficiency, but that the orientation of the G-C base-pairs appear to be of little importance (Figure 7).” And we have also added the following sentence to the discussion: “The downstream hairpin plays a key role, since base-pairing in one part of its stem is essential for efficient cleavage (Figure 7), whereas other specific sequence elements (but not their base-pairing) are important for positioning the cleavage (Figure 8).”

Line 683: It remains speculative why and how this upstream sequence (sectorII) would be important. Is a similar upstream region also important in the other target genes.

Reply: We would like to thank Reviewer 1 for this question, which has led us to test (and correct) one of the interpretations of the study.

Indeed, the only data we had regarding sector II was that deleting the sector led abolished the cleavage, which does not provide a lot of detail on the mechanism. To answer this question from reviewer 1, we have generated a range of pSaGap mutants where sector II has been mutated (rather than deleted). To our surprise, all four mutant transcripts were cleaved by RNase Y! (please see Supplementary Figure S5), meaning that any random nucleotides (or at least those we tested) can be upstream of the G, but that moving sector I close to the cleavage site abolishes cleavage. This is somewhat mysterious, since removing both sector I and sector II does not re-instate the cleavage (Figure S5A).

Furthermore, to determine whether an upstream deletion would prevent cleavage in other transcripts, we deleted the 12 nucleotides upstream of the conserved G in pBsCgg and pBsGln. Once again, we observed cleavage of the mutated transcripts, leading us to the conclusion that sector II is only important for the gap transcripts, and then only if it is deleted, not when its sequence is merely changed (please see Supplementary Figure S5).

We have added the following paragraph to the manuscript to describe our findings:

“The role of sector II was further explored by generating four mutated versions of sector II, all of which retained the G residue: SecIImutant1, where the 12 nucleotides from the hairpin of sector I replaced the 12 nucleotides of sector II; SecIImutant2, where sector II was replaced by the 12 equivalent nucleotides from the SaGln transcript; SecIImutant3, where the first half of sector II was mutated and SecIImutant4, where the second half of sector II was mutated (S5C Fig). All four of these mutant transcripts were cleaved in an RNase Y-dependent manner (S5D Fig), suggesting that sector II must be present for cleavage to occur, but that its sequence is of little to no importance. To determine whether a similar deletion would have the same effect on other transcripts, we deleted the 12 nucleotides upstream of the conserved G residue of pBsCgg and pBsGln (generating pBsCgg[Δ12] and pBsGln[Δ12], respectively), and observed that both of the mutant transcripts were cleaved by RNase Y, although the efficiency of cleavage appears to be lower in pBsGln[Δ12] (S5E Fig and S5F Fig).”

We have modified the discussion and our conclusions to include these new data.

Line 725: Show the predicted structures of all 4 transcripts.

Reply: The predicted structure of pSaGap is outlined in Figure 4 and we have now added a figure with the predicted structures of pSaGln, pBsCgg and pBsGln (Figure S9).

Reviewer #2: RNase-driven decay of RNA is a universal and essential process for all living organisms that rapidly adapts transcript availability to current needs and also mediates the processing and maturation of cellular RNAs. The present study focusses on the endoribonuclease RNase Y of the Gram-positive bacterium S. aureus and the factors determining its cleavage function.

The authors opted for an in vivo system in which a native S. aureus and a corresponding RNaseY deletion mutant were used as the experimental backgrounds for cleavage of model transcripts provided on a vector. In an impressive series of elegantly conceived and meticulously conducted experiments, the authors identified the sequence and structural constraints that determine RNase Y target site selection and cleavage efficiency. Also, they demonstrate that RNases Y from S. aureus and B. subtilis can functionally replace each other in vivo. This is a comprehensive study, exhaustive down to the smallest detail, which builds on much previous knowledge to provide an accurate picture of how RNase Y selects and cleaves its RNA targets.

The manuscript is very well written and leads the reader logically and step by step through the experiments and their theoretical considerations as well as the results. I would like to emphasise that it was a real pleasure to follow this scientific endeavour. However, this strength of the manuscript is also its weakness.

1. Although all data and details were obtained and can be found in the paper, they are not summarised at any point in a concise and comprehensible manner. The authors should definitely look for a way to rectify this, for example by revising the abstract and also by adding a section (either in the results section or the discussion) in which they clearly identify the sequence and structural features that determine RNaseY targets. Figure 10 in the Discussion would be a good starting point to build on. Otherwise, there is a risk that the data cannot be adequately utilised by the scientific community.

Reply: We have modified the abstract to provide more detail about our findings, and we have reorganised the last part of the discussion in order to lead up to a final conclusion that includes an overview of the findings. For this we have added the following paragraph to the discussion: “Taken together, our data shows that the region surrounding the RNase Y cleavage site in SaGap has all the factors required for both targeting by RNase Y and cleavage at a precise position. Moreover, different elements control the cleavage efficiency and the cleavage positioning. The downstream hairpin plays a key role, since base-pairing in one part of its stem is essential for efficient cleavage (Fig 7), whereas other specific sequence elements (but not their base-pairing) are important for positioning the cleavage (Fig 8). Only the length of the sequence between the cleavage position and the hairpin is important for positioning (Fig 4), but its composition can modify the cleavage efficiency more than 20-fold (Fig 9). While the SaGap transcript was the main focus, we have demonstrated several of the above-mentioned elements in other transcripts from both S. aureus and B. subtilis (summarised in Fig 10).”

We have furthermore modified Figure 10 to give a better overview and to underline which of our findings appear to be specific to SaGap and which are more generally applicable.

More specifc comments:

2. Figure 1D: Is this deceptive or does the pSaGap allow a little more leeway regarding the cleavage site than the other transcripts? It seems that there are a little bit more alternative cleavage sites (?)

Reply: We have noticed the same thing, and it is in particular a leeway of a single nucleotide towards the downstream direction. We think that it is due to SaGap being the only one of the four transcripts to have a purine (an A in this case) immediately downstream of the main cleavage site. If the molecular ruler is slightly imprecise (and we show with deletion of sector III that it is), and if at the same time there is a purine, then RNase Y might in some cases cleave at the alternative position. Transcripts SaGln, BsCgg and BsGln all have the non-preferred U’s or C’s just after the main cleavage, and RNase Y will therefore not find a preferred cleavage position immediate next to the main cleavage in these three transcripts.

3. Figure 4 B and C, last panel (pSaGap sector V/VI deletion). In the Northern blot, cleavage is obviously impaired in this mutant, but the EMOTE data (Fig 4 D) still show cleavage at the correct position. How can this be explained?

Reply: Upon re-reading the manuscript we now realise that while we do mention this discrepancy in the text, we do not provide a possible explanation. Further in the same section we suggest that similar results observed for pSaGap[ΔIV] and pSaGap[ΔII+G] are due to partial redundancy with other sectors. We have re-organised the section to better make the link between these similar results.

Moreover, we have added a sentence to explain why the EMOTE results from transcripts which are not even visible in a Northern can result in such a high column in our EMOTE column diagrams: “Note that the EMOTE data is presented as the number of detected molecules with a given 5’-end divided by the total number of detected molecules within the chosen window. The vertical axis therefore does not reflect the total intracellular concentration of molecules with a given 5’-end, but rather its abundance relative to the other molecules in the chosen window.”

4. pg. 13, experiment on the exogenous fliM transcript: "We could detect the full-length fliM transcript in both S. aureus WT and delta Y strains". In Figure 5B left, I can only see a signal in the RNase Y mutant background, but not in the wildtype.

Reply: The band is visible on our pdf document (although it is weaker that for the deltaY strain). We have tried to adjust the contrast on the image to better show the band, please let us know if the problem persists.

5. Further to the experiment on the exogenous fliM transcript: Did actually the surrounding fliM RNA of the gap II-V insertion influence the folding of the RNase Y target?

Reply: The question is very relevant but difficult to answer, since changes in any nucleotide sequence of an RNA can alter the secondary structure in ways that are not readily predictable in silico. Furthermore, any RNA sequence of more than a few nucleotides will be predicted to form secondary structures (whether these form in real-life or not).

However, in this case when we use mFold to predict the structures upstream of the cleavage sites in the fliM::gapII-V transcript, it predicts a strong hairpin ending 20 nucleotides upstream of the cleavage sites (i.e. outside of the gap-insert). This hairpin does appear to attract RNases since the un-altered fliM transcript is not cleaved.

When we use mFold on a 200 nt RNA sequence with the two cleavage sites in the middle, we obtain extensive predicted duplex formations, but with very short/weak duplexes near the cleavage sites (although the sector IV hairpin is predicted to be conserved). These predictions were not part of the original submission, since the identity of the unknown RNase is not the subject of this paper.

We have added the predicted structure in a new supplementary figure (Figure S9A).

6. pg. 20 line 577 "The wild-type trinucleotide sequence (AGA) turned out to be one of the worst sequences for RNase Y cleavage efficiency". Did this sequence evolve/prevail to protect S. aureus RNAs from excessive RNA cleavage? If so, why are other poor-cleaving triplets under-represented as targets?

Reply: We suspect that the gap transcript evolved to be cleaved and that there is a continuous evolutionary pressure to maintain the RNase Y site. However, there is also an evolutionary pressure to prevent every single gap mRNA from being cleaved (since that would lead to lack of gapR). The AGA trinucleotide is one way of accomplishing this equilibrium (although evolution could presumably equally well have ended up with one of the other somewhat poorly cleaved tri-nucleotides such as GGA or CGA).

Each S. aureus transcript will have its own optimal cleavage frequency and the genome has presumably evolved to accommodate this optimisation (for example, the SaGln transcript has a CCA trinucleotide).

Reviewer #3: The ribonuclease RNase Y is a player in RNA metabolism in Firmicute bacteria, and in this study, the authors have explored the determinants for RNase Y activity on target RNAs. The authors show that the nucleotide sequence surrounding an RNase Y cleavage site is sufficient for cleavage and demonstrate that a non-substrate RNA can be converted into a substrate. They use mutational analyses to determine which nucleotides in the vicinity of the cleavage are required for efficient cleavage and which nucleotides determine the exact cleavage position. These findings are shown to operate in both S. aureus and B. subtilis and are likely to be general within the Firmicutes. They present evidence that the S. aureus gapR transcript has evolved to maintain a precise cleavage position, but with low cleavage efficiency, and that the efficiency can be modulated by sequence changes in the RNA. One striking result is that they mapped the RNase Y recognition within the GapR operon and showed that this region can be inserted into a different RNA molecule and convert it into an RNase Y target. The study is well conducted and the results clearly explained.

Reply: Thank you.

The authors mainly utilized in vivo approaches, such as Northern blotting, and EMOTE, to elucidate the mechanism of RNase Y cleavage site and the secondary structural requirement of transcripts for the catalysis. The results and reasoning are mostly clear and understandable, but in vitro experiments (in the future) might be also helpful to support some of their conclusions. There are a few other minor comments that will hopefully be helpful for the authors to consider:

Interesting new reference with might be added at line 37? Jenniches et al (2024) Improved RNA stability estimation through Bayesian modelling reveals most Salmonella transcripts have subminute half-lifes. 121, e2308814121.

Reply: Thank you for drawing our attention to this highly interesting study. The reference has been added and the introduction has been changed according to the new information.

Line 144 ‘argued’ might be better replaced with ‘reasoned’?

Reply: We agree. The replacement has been done.

Page 11, line 328-329

Investigation of mutation in conserved bases in the sector II between B. subtilis and S. aureus would be interesting. It might be interesting if the authors can try test cleavage pattern with a simplified system in vitro.

Reply: Our choice of examining the RNase Y cleavage in vivo was to ensure that all required co-factors, salt-concentration, enzyme/substrate concentrations and stoichiometry, etc. would be in place. Moreover, like the majority of RNase Y targets, the model targets we are studying are all Ric-complex dependent (the manuscript describing this was published after we had done a large part of the work). An in vitro system would therefore need to include RicT, and possibly even RicA and RicF to load RicT onto RNase Y.

Nevertheless, we fully agree that an in vitro system could shed light on some of the important details of the RNase Y target selection and positioning. However, we do not currently have such a system in place in our lab.

Page 11, line 337-339

The disappearance of cleaved band caused by the truncation of sector V and VI may also be merely due to the lack of scaffold for RNase Y to be recruited on the RNA. This can be tested by substituting these sectors with a long nucleotide which does not form any secondary structures.

Reply: The transcript does not end at the 3’end of sector VI, but continues for another 257 nucleotides, so RNase Y should in principle have enough scaffold for its recruitment.

If we put these 257 nt into mFold we will of course get some predicted structures (as with any stretch of random RNA), but the only structure we are sure about is the rho-independent terminator at the extreme 3’-end.

Page 13, line 390-394

It is very interesting that an exogenous RNA (E. coli fliM) could be transformed into a target for RNase Y by adding regions II-V of SaGap. Is there a hypothesis on what other endoribonuclease might be getting recruited and cleaving in the vicinity and why this is not the case in SaGap?

Reply: We do not know which RNase performs the “new” cut. We tried to test if it was perhaps RNase J1, but unfortunately the transcripts are not expressed (or at least so poorly that we cannot detect them) when we transform our vectors into an RNase J1 mutant. It does not appear likely to be RNase III, since we do not predict any significant additional secondary structures when combining fliM with the 103 nt of gap.

Does the negative control using the RNase Y knockout strain in this experiment eliminate the possibility that an unknown ribonuclease cleaved the transcript from pfliM::II-V at both canonical and 2nt upstream of canonical sites instead of RNase Y ?

Could an unknown nuclease work dependently on RNase Y?

Reply: Indeed, we cannot formally exclude that both of the observed cleavage events are due to the same unknown RNase, and that one of the cleavages by this unknown RNase is dependent on the presence of RNase Y. We find such an explanation unlikely due to the number of coincidences that would have to co-occur for this to happen, but we have nevertheless used the term “RNase Y dependent cleavage” (instead of “cleavage by RNase Y”) to indicate the possibility of another endonuclease.

Page 15, line 394

The reference of the figure should be 3D.

Reply: Corrected.

Page 18, line 542

The numbering of mutated base (U280A) does not match with that displayed in the figure 8A (U282A). This is the case with other sentences in this manuscript.

Reply: The numbering in figure 8 was incorrect. It has been corrected.

Page 23, line 684-689

Could deletion of both sector I and II result in putting another hairpin structure (which may be formed the sequence upstream of these sectors) in proximity to the cleavage site and thereby occluding it? This may need to be tested and mentioned in this article.

Reply: We did take that possibly into account when we generated our mutants, and mFold was unable to predict any convincing structures at the relevant position of the sectorI+II deletion transcript. However, this information was indeed not included in the original manuscript, and what constitutes “convincing” is obviously subjective. We have therefore now added a supplementary figure and the following sentence to the manuscript: “Here it should be noted that the deletion of both sectors I and II will place a very weak predicted hairpin structure close to the cleavage site (S5B Fig), which theoretically could be the factor that blocks cleavage.”

Page 25, line 765-768

This explanation of why the precise cleavage can be advantageous might need rephrasing for clarity.

Reply: We have reformulated the sentence, and it now reads: “Such precision is presumably of little importance if the cleavage products are destined for immediate degradation, but precision might be advantageous in situations where one or both of the two resulting RNA cleavage products should have ends that protects them against exoribonucleases.”

Figure 5: The levels for fliM in the WT background seem lower than in the ΔY strain.

Reply: It is indeed a weaker signal. It is possible that RNase Y participates (directly or indirectly) in a degradation pathway of fliM, without leading to degradation fragments that detectable by Northern blot (or at least not with the probe that we used).

Figure 8 and lines 541-544: Can the order of the A264U and U282A mutants in Figure 8 be swapped to match the order they are mentioned in the text?

Reply: Done

Figure 9: Could they highlight those trinucleotide combinations that are mentioned in the text?

Reply: Done

Have all data underlying the figures and results presented in the manuscript been provided?

Large-scale datasets should be made available via a public repository as described in the PLOS Genetics data availability policy, and numerical data that underlies graphs or summary statistics should be provided in spreadsheet form as supporting information.

Reviewer #1: Yes

Reviewer #2: Yes

Reviewer #3: Yes

PLOS authors have the option to publish the peer review history of their article (what does this mean?). If published, this will include your full peer review and any attached files.

If you choose “no”, your identity will remain anonymous but your review may still be made public.

Do you want your identity to be public for this peer review? For information about this choice, including consent withdrawal, please see our Privacy Policy.

Reviewer #1: No

Reviewer #2: No

Reviewer #3: No
